# Navigating the Cervico-Axillary Canal: A Rare Encounter With Double Dumbbell Space Sarcoma Involving the Neck and Axilla

**DOI:** 10.7759/cureus.54921

**Published:** 2024-02-26

**Authors:** Saheer Neduvanchery, Ravindran Chirukandath, Sumin V Sulaiman, Ireddy Sandeepreddy, Priyanka Mittra

**Affiliations:** 1 Surgical Oncology, Government Medical College, Thrissur, IND; 2 General Surgery, Government Medical College, Thrissur, IND

**Keywords:** lipo sarcoma, space sarcoma, sarcoma, dumb bell, cervico axillary canal

## Abstract

Space sarcomas are exceedingly rare neoplasms, and double dumbbell space sarcoma in the cervicoaxillary canal has not been previously reported. We present a case of a 63-year-old male who presented with a swelling in the neck and axilla of four years' duration, which rapidly increased in size over the last three months. Clinical examination and imaging revealed a multiseptate mass extending from the posterior triangle of the neck to the right axilla and chest wall through the cervicoaxillary canal. This lesion encased major vessels and components of the brachial plexus but did not infiltrate them. A trucut biopsy confirmed the diagnosis of well-differentiated liposarcoma. Surgical intervention was performed, achieving complete resection with preservation of neurovascular structures. This case highlights the unique challenges and complexities associated with managing double dumbbell space sarcomas in the cervicoaxillary canal. Additionally, it underscores the importance of a multidisciplinary approach to achieving successful outcomes while preserving limb function and minimizing complications. Long-term follow-up is essential for monitoring potential recurrences.

## Introduction

Liposarcoma stands as one of the most prevalent soft-tissue sarcomas found in adults, making up approximately 20% of all soft-tissue malignancies [1]. Typically, its occurrence is observed in the retroperitoneum and lower extremities, particularly within structures like the intermuscular fascia [2]. Extracompartmental space sarcoma is rare, and we present an unusual occurrence of liposarcoma in the neck and axilla that arose from a pre-existing, long-standing lipoma. At the outset, individuals diagnosed with well-differentiated liposarcomas in their extremities typically have a more favorable prognosis. As a result, the primary treatment approach should involve wide excision. Even in situations where the lesions are in close proximity to major nerves or blood vessels, conservative resection techniques that preserve these crucial structures and maintain limb function have demonstrated remarkable effectiveness, resulting in minimal recurrence and metastasis rates [3].

## Case presentation

**Figure 1:**
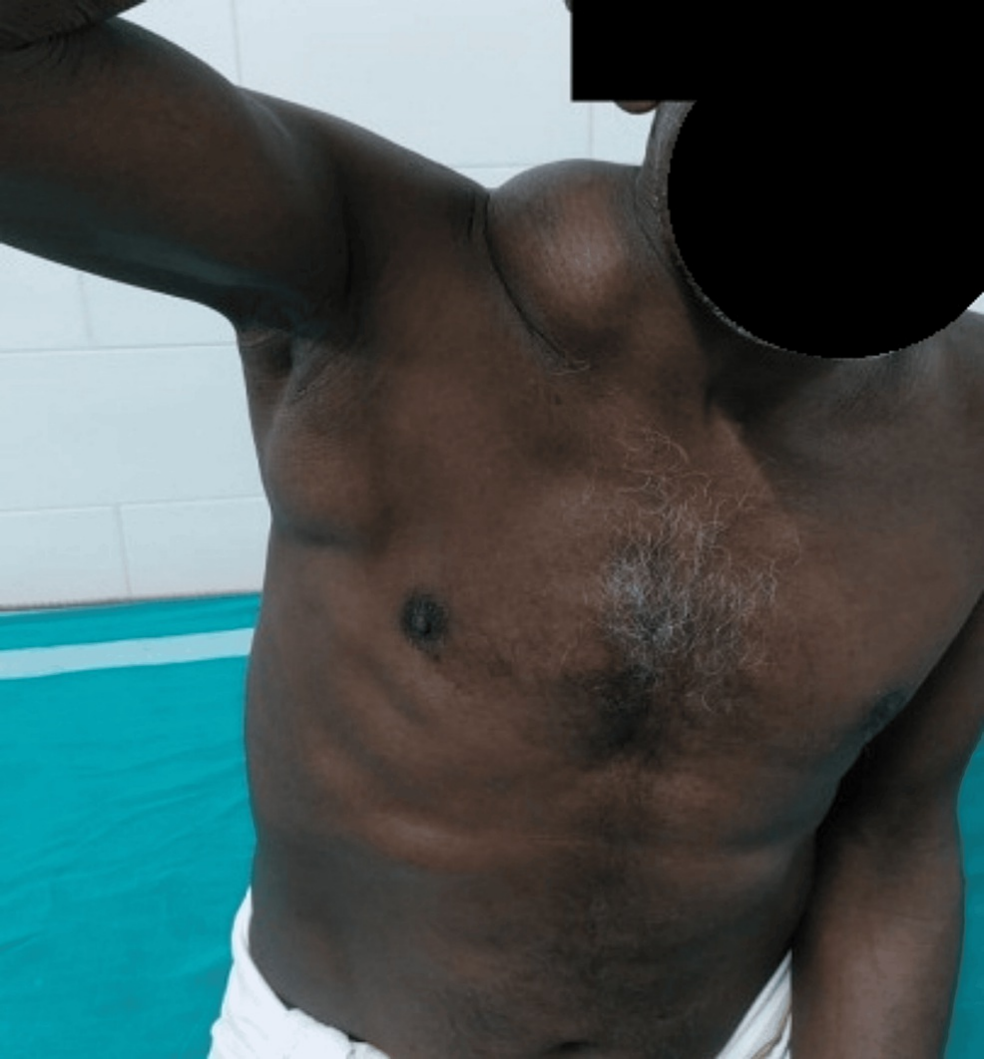
Dumb bell tumor in neck and axilla

A 63-year-old gentleman presented with a swelling in his right neck and axilla, which had been persisting for four years but had significantly increased in size over the last three months. The patient had no prior history of comorbidities or surgical procedures. Physical examination revealed a distinctive dumbbell-shaped mass measuring 20 cm × 15 cm in the root of the neck and 15 cm × 10 cm in the right axilla (Figure 1). The neck lesion was mobile and displayed varying consistency with both soft and hard areas, while the chest lesion was subpectoral. There was no palpable axillary lymph node enlargement, focal neurological deficits in the right arm, or muscle weakness.

**Figure 2:**
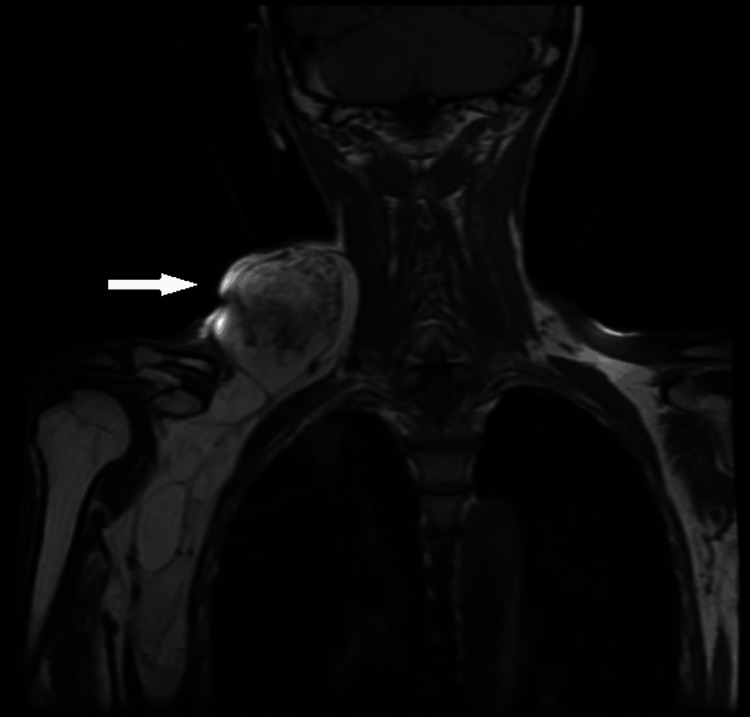
MRI showing space sarcoma in the cervicoaxillary canal

An MRI scan demonstrated a 24 cm × 18 cm × 11 cm multiseptate lesion extending from the posterior triangle of the neck to the right axilla and chest wall through the thoracic inlet (Figure 2). The lesion abutted the internal jugular vein superiorly, the trapezius muscle posteriorly, the paraspinal muscles medially, and the sternocleidomastoid muscle laterally, maintaining fat planes. Notably, the lesion encased the subclavian vessels and components of the brachial plexus without evidence of infiltration.

A trucut biopsy performed on solid areas of the lesion suggested a diagnosis of well-differentiated liposarcoma. Given the rarity of double dumbbell space sarcomas and the unique location and relationship of this lesion with major vessels, surgical intervention was deemed necessary.

**Figure 3:**
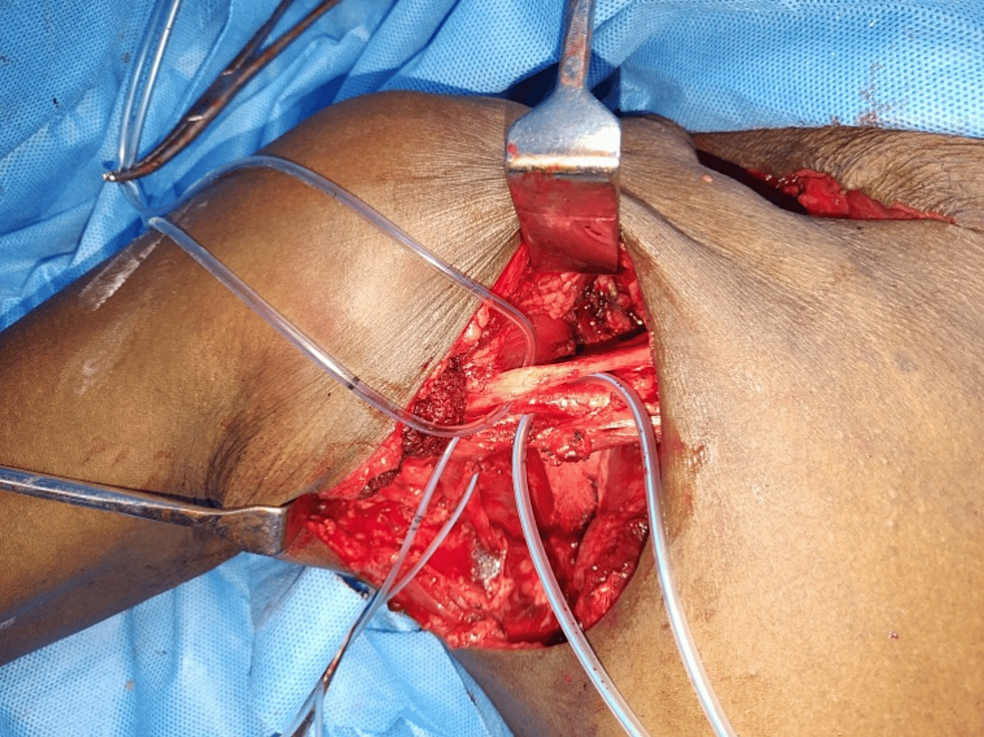
Taped brachial plexus and subclavian vessels

**Figure 4:**
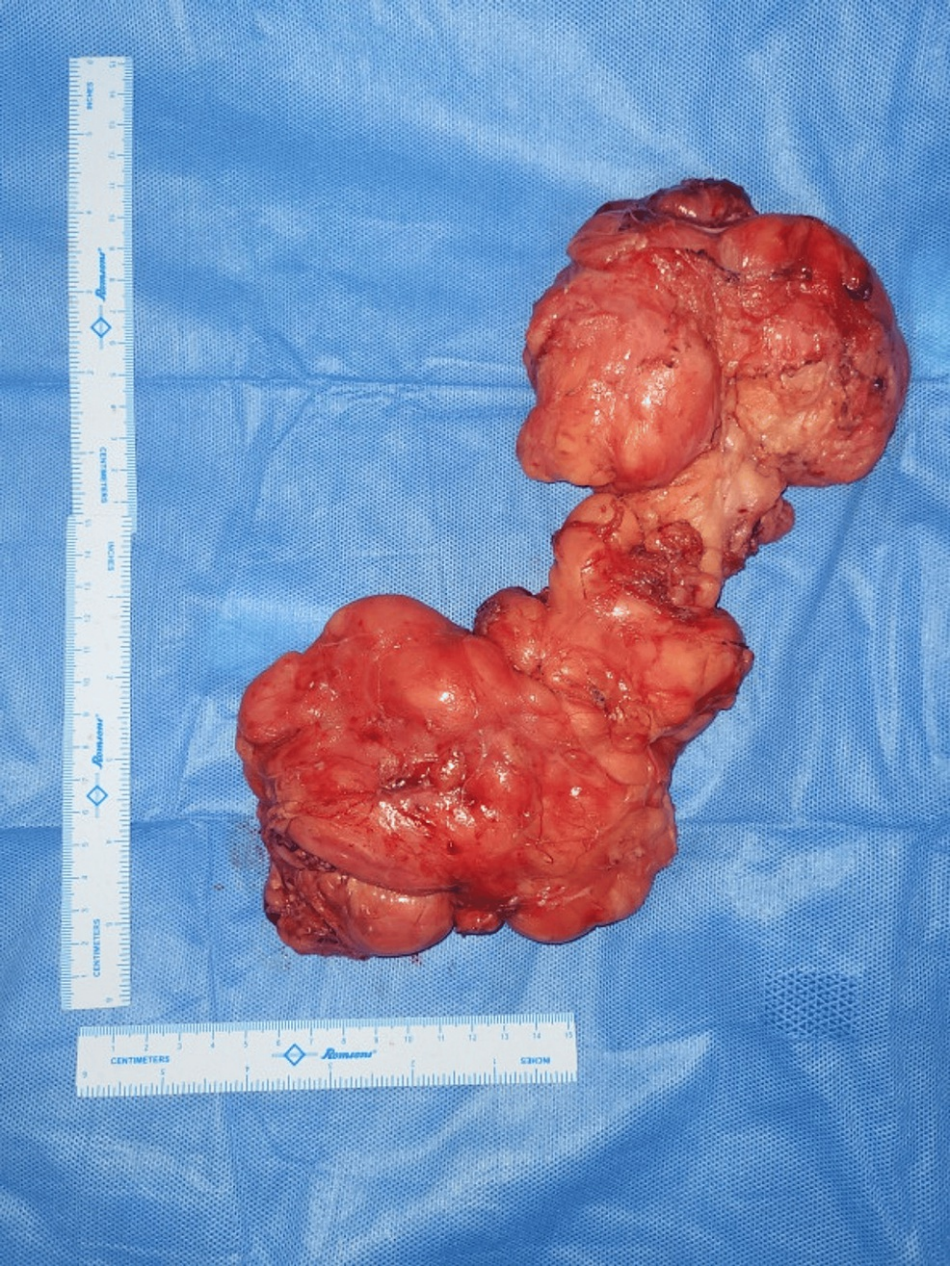
Dumbbell tumor

**Figure 5:**
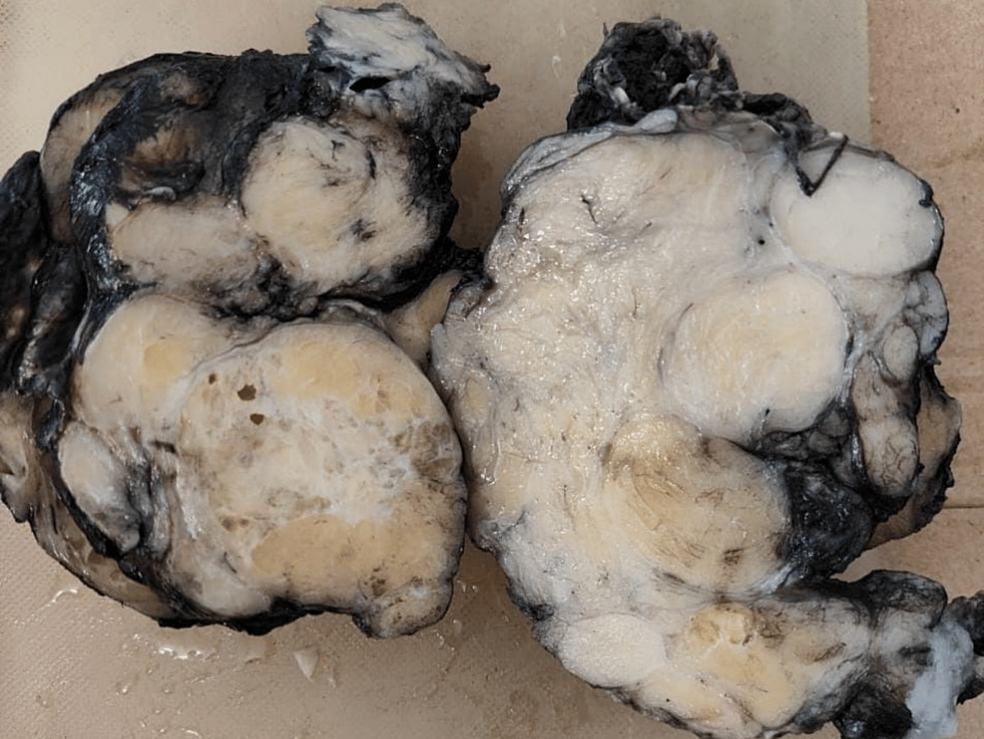
Gross image of the lesion Cut surface showing yellow, fatty tissue with interspersed fibrous septae, areas of necrosis, and hemorrhage

**Figure 6:**
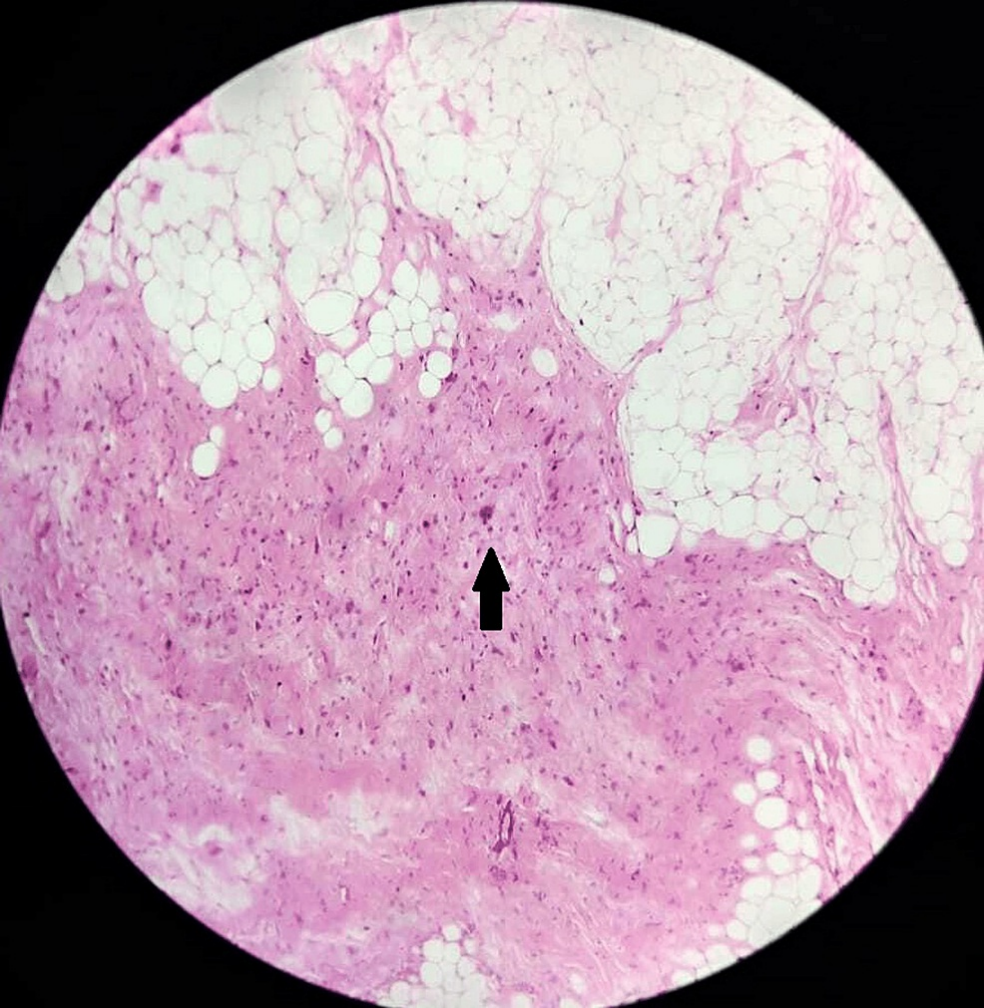
Microscopy of the lesion Microscopy showing adipocytes with hyperchromatic pleomorphic nuclei and multinucleated tumor cells

Surgical excision was undertaken using two incisions, one in the neck and another in the axilla. The lesion was first mobilized from the neck, dissecting it from the trapezius, sternocleidomastoid, and paraspinal muscles. Subsequently, through the axillary incision, the axillary part of the lesion was mobilized after careful taping of the brachial plexus and subclavian vessels (Figure 3). An extracapsular dissection was performed without tumor spillage, preserving the integrity of vessels and nerves and achieving an R0 resection (Figure 4). Postoperatively, the patient did not display any neurovascular deficits or weakness. The dumbbell tumor specimen submitted for histopathological examination exhibited the gross characteristics of a soft, fleshy mass with extensive areas of hemorrhage and necrosis (Figure 5). Microscopic analysis revealed liposarcoma, manifested by sheets of large pleomorphic cells featuring vesicular hyperchromatic nuclei, prominent mitotic activity, and infiltration into the thick capsule, indicative of the tumor's aggressive nature (Figure 6).

## Discussion

The thoracic outlet is precisely defined as the region in the lower neck situated between the thorax and axilla, serving as the conduit through which the subclavian vein, subclavian artery, and brachial plexus traverse from their central origins to their peripheral destinations. This anatomical region is demarcated by the clavicle anteriorly, the first thoracic rib posteriorly, the insertion point of the pectoralis minor muscle, and the humerus laterally. Functionally, it is partitioned into three distinct areas: the scalene triangle situated above the clavicle, the costoclavicular space, or cervicoaxillary canal, located between the clavicle and the first rib, and the subcoracoid or pectoralis minor space positioned below the clavicle [4,5]. This spatial zone encompasses the primary subclavian artery, subclavian vein, and brachial plexus. This is a narrow and confined space, and any space-occupying lesion within this canal presents a significant challenge for surgical removal.

Soft tissue sarcomas (STS) originating in extra-compartmental spaces exhibit distinctive features. These tumors possess the capacity to extend over a significant distance both proximally and distally, encountering fewer anatomical constraints. As a consequence, tumors arising within these spaces tend to be closely situated within the neurovascular bundle, often leading to early involvement [6-8]. The cervicoaxillary canal's limited dimensions make the removal of any space-occupying lesion within it a demanding surgical task.

Wide excision surgery produces excellent results in the treatment of massive liposarcomas of the extremities in terms of not only a low recurrence rate but also good limb function [9]. Space sarcomas involving the cervicoaxillary canal are exceptionally uncommon. The location of this lesion, with its proximity to major vessels and the brachial plexus, adds a significant level of complexity and interest to the case. A trucut biopsy performed on solid areas of the lesion suggested a diagnosis of liposarcoma, prompting surgical intervention.

The objectives of resection for soft tissue sarcomas in the extremities encompass achieving a wide excision of the tumor with clear, negative resection margins, all while preserving the optimal function of the affected limb. Intra-compartmental tumors typically entail resection of both the tumor and the surrounding muscle to accomplish this goal. In contrast, space tumors, despite their close proximity to blood vessels, may sometimes be resected with negative margins while sparing the neurovascular bundle. However, in other instances, the behavior of these tumors may lead to vascular involvement, necessitating their resection in accordance with the extent of vessel involvement [10].

## Conclusions

An understanding of the anatomy of the cervicoaxillary canal is essential for diagnosing and managing conditions such as double dumbbell space sarcoma. The precise location and relationships of structures within this anatomical region can greatly impact surgical planning and outcomes. Careful dissection, preservation of neurovascular structures, and attention to maintaining fat planes are crucial aspects of successful management in this challenging anatomical space.
